# Azithromycin-Loaded Nanoparticles Incorporated in Chitosan-Based Soft Hydrogels: A Novel Approach for Dental Drug Delivery

**DOI:** 10.3390/pharmaceutics17030304

**Published:** 2025-02-26

**Authors:** Jakub Kwiatek, Magdalena Paczkowska-Walendowska, Anna Rył, Tomasz M. Karpiński, Andrzej Miklaszewski, Ewelina Swora-Cwynar, Marta Leśna, Judyta Cielecka-Piontek

**Affiliations:** 1 Kwiatek Dental Clinic Sp. z o.o., Kordeckiego 22, 60-144 Poznan, Poland; jakubkwiatek@klinikakwiatek.pl (J.K.); martalesna@klinikakwiatek.pl (M.L.); 2 Department of Pharmacognosy and Biomaterials, Poznan University of Medical Sciences, Rokietnicka 3, 60-806 Poznan, Poland; jpiontek@ump.edu.pl; 3 Science-Bridge Sp. z o.o., Chociszewskiego 24/8, 60-258 Poznan, Poland; 4 Department of Chemical and Molecular Engineering, Lodz University of Technology, Wolczanska 213, 93-005 Lodz, Poland; anna.ryl@p.lodz.pl; 5 Department of Medical Microbiology, Medical Faculty, Poznan University of Medical Sciences, Rokietnicka 10, 60-806 Poznan, Poland; tkarpin@ump.edu.pl; 6 Faculty of Materials Engineering and Technical Physics, Institute of Materials Science and Engineering, Poznan University of Technology, 60-965 Poznan, Poland; andrzej.miklaszewski@put.poznan.pl; 7 Department of Pharmacology and Phytochemistry, Institute of Natural Fibres and Medicinal Plants—National Research Institute, Wojska Polskiego 71b, 60-630 Poznan, Poland; eswora@ump.edu.pl

**Keywords:** azithromycin, chitosan hydrogels, drug delivery, controlled release, mucoadhesion, antioxidant activity, anti-inflammatory properties, antimicrobial activity, dental applications

## Abstract

**Background:** Azithromycin (AZC), a BCS class II/IV antibiotic with broad-spectrum antimicrobial activity, has poor water solubility, limiting its formulation potential. This study aimed to develop and optimize AZC-based soft hydrogels for the first time for improved solubility, local controlled drug release, and local dental applications. **Methods:** AZC nanoparticles (based on polyvinylpyrrolidone) were synthesized via electrospinning enhanced solubility 40-fold. These were incorporated into chitosan (CS) hydrogels with varying concentrations and degrees of deacetylation (DDA), optimized using a factorial design. Hydrogels were characterized for drug release, mucoadhesion, antioxidant, anti-inflammatory, and antimicrobial properties, with Principal Component Analysis (PCA) assessing correlations. **Results:** Soft hydrogels with 3% CS and 80% DDA achieved sustained drug release (62.9–94.7% over 48 h), strong mucoadhesion, and enhanced biological activity. Higher CS and DDA improved antioxidant and anti-inflammatory effects due to increased free amino groups. Antimicrobial tests showed efficacy against *Streptococcus mutans* and *Staphylococcus aureus*. PCA revealed an inverse correlation between AZC release and mucoadhesion and positive correlations between release and anti-inflammatory activity. **Conclusions:** AZC-based soft hydrogels significantly improved solubility, controlled release, and biological activity, showing strong potential for dental drug delivery. Further clinical validation and optimization are recommended.

## 1. Introduction

Azithromycin (AZC) is a macrolide antibiotic known for its broad-spectrum activity against various bacterial infections. It demonstrates bacteriostatic effects against various oral microorganisms, including anaerobic and Gram-negative bacteria [1,2]. However, despite its effectiveness, AZC faces significant formulation challenges due to its poor water solubility and, in some cases, limited bioavailability, as it is classified as a Biopharmaceutics Classification System (BCS) class II or IV compound [3]. These limitations restrict its therapeutic potential, particularly for localized treatments such as oral infections, where sustained and targeted drug release is crucial. Currently, oral administration is the recommended method of use [4]. A key issue with systemic antimicrobials for dental infections is patient compliance, highlighting the need to develop localized drug delivery systems [1]. Currently, the only available dental drugs are gels containing metronidazole or framycetin.

Hydrogels have emerged as versatile drug delivery systems capable of addressing solubility and release challenges [5]. These three-dimensional, hydrophilic polymer networks can absorb significant amounts of water, creating an ideal environment for controlled drug release [6]. They are frequently employed as delivery vehicles for tissue engineering [7], oral tissue regeneration, and oral mucosal drug delivery [8] due to the prolonged residence time on the mucous membrane compared to rinses. Chitosan (CS), a natural biopolymer derived from chitin, has attracted considerable attention in hydrogel development due to its biocompatibility, biodegradability, and antimicrobial properties [9]. Using its mucoadhesive properties, CS extends the residence time and, consequently, the action time at the application site [10].

Additionally, the degree of deacetylation (DDA) of CS, which determines the proportion of free amino groups (-NH_2_), significantly impacts its physicochemical and biological properties [11]. Adjusting the DDA and concentration of CS in hydrogels provides a mechanism to modulate key features such as drug release rate, bioadhesion, and antioxidant and anti-inflammatory activities. CS has been utilized in dentistry for caries prevention, nano-materials, and CS-based hydrogels to increase mechanical integrity, antimicrobial previously damaged tissue regeneration, dentin matrix, and to close the canal space during root canal therapy [12,13].

There have been isolated reports of efforts to develop forms specifically designed for local application. Incorporating AZC into thermosensitive chitosan-based hydrogels demonstrated significant potential for use in regenerative endodontic procedures, enhancing antibacterial efficacy and promoting tissue repair in an in vivo model [14]. Kerdmanee et al. developed the thermoresponsive AZC-loaded niosome gel with sustained drug release and enhanced antimicrobial activity [15]. Meanwhile, Ayoub et al. produced photocrosslinkable AZC-laden gelatin methacryloyl fibers via electrospinning with adequate mechanical and degradation properties [16]. Finally, Abruzzo et al. developed azithromycin-loaded liposomes and niosomes to treat topical infections [17], which require deep tissue penetration or intracellular delivery. The indicated forms are only delivery systems that must be incorporated into the appropriate matrix for further application.

Integrating AZC nanoparticles into chitosan-based hydrogels presents a novel approach to improving solubility, enabling localized and sustained delivery in dental applications. Such hydrogels could provide therapeutic advantages, including prolonged retention at the application site, controlled drug release, and synergistic antimicrobial and anti-inflammatory effects. These properties are particularly relevant in combating dental infections, often involving persistent biofilms and inflammation. The oral cavity presents a complex and dynamic environment, where factors such as saliva flow, mechanical forces from chewing, and microbial diversity pose challenges for effective treatment. Hydrogels, with their ability to adhere to oral tissues and provide sustained release of therapeutic agents, offer a promising solution for addressing these challenges.

Furthermore, hydrogels can be designed to deliver antimicrobial, anti-inflammatory, or antioxidant agents directly to affected sites, helping to manage oxidative stress and microbial resistance—two critical concerns in oral health [18]. Their tunable degradation rates and ability to form protective barriers also make them ideal for prolonged action at the site of application [6]. They enhance therapeutic outcomes by providing controlled and localized drug delivery while minimizing systemic side effects. They are well-suited for treating conditions such as periodontitis, dental caries, and post-surgical healing [19].

This study aimed to develop AZC-based nanoparticles with enhanced solubility for the first time and incorporate them into soft hydrogels with varying CS concentrations and DDA levels. Polyvinylpyrrolidone (PVP) was selected to prepare nanoparticles, a biocompatible, water-soluble polymer known for its excellent stability and ability to encapsulate and protect active agents. Its role in forming nanoparticles ensures efficient drug loading and sustained release, which is crucial for prolonged antimicrobial and anti-inflammatory effects in the oral cavity [20]. On the other hand, chitosan was chosen as the hydrogel matrix due to its well-documented bioadhesive, antimicrobial, and biocompatible properties. Combining PVP-based nanoparticles within a chitosan hydrogel creates a synergistic system that ensures controlled drug delivery while enhancing adhesion and efficacy in the complex oral environment. The design of the experiment (DoE), particularly a full factorial design, was employed to optimize the hydrogel formulations. The soft hydrogels were evaluated for key properties, including drug release behavior, mucoadhesion, antioxidant and anti-inflammatory activities, and antimicrobial efficacy. The findings of this research are expected to contribute significantly to the field of dental therapeutics by providing a robust platform for localized drug delivery. The potential of AZC-based soft hydrogels to address common dental issues, including infections and inflammation, underscores their relevance in advancing oral healthcare. This work lays the foundation for further optimization and clinical validation of chitosan-based hydrogels as practical and versatile drug delivery systems for AZC and other therapeutic agents requiring enhanced solubility and targeted delivery.

## 2. Materials and Methods

### 2.1. Chemicals

Azithromycin dihydrate (AZC) was obtained from TriMen Chemicals Sp. z o.o. (Łódź, Poland), while Azithromycin dihydrate as European Pharmacopoeia (EP) Reference Standard from Sigma-Aldrich (Poznan, Poland). Excipients, such as chitosans (with deacetylation degree DDA < 67.5–72.5% ≈ 70% and viscosity of 1% in 1% acetic acid 351–750 mPas, DDA 77.6–82.6% ≈ 80% and viscosity of 1% in 1% acetic acid 351–750 mPas, DDA 87.6–82.5% ≈ 90% and viscosity of 1% in 1% acetic acid 351–750 mPas) were supplied from Heppe Medical Chitosan GmbH (Halle, Germany), while polyvinylpyrrolidone (PVP) K30 was supplied from Sigma-Aldrich (Poznan, Poland). Reagents for activity assays (2,2-Diphenyl-1-picrylhydrazyl (DPPH), sodium chloride, bovine serum, hexadecyltrimethylammonium bromide (CTAB), hyaluronic acid (HA)), dissolution studies (potassium chloride, sodium chloride, di-potassium hydrogen ortho-phosphate, magnesium chloride, calcium chloride, xylitol) and bioadhesive tests (mucin from porcine stomach) were obtained from Sigma-Aldrich (Poznan, Poland). The HPLC-grade acetonitrile and water were obtained from Merck (Darmstadt, Germany). High-quality pure water and ultra-high-quality pure water were prepared using a Direct-Q 3 UV Merck Millipore purification system.

### 2.2. Preparation of AZC-Based Nanoparticles

Nanoparticles were prepared using the electrospinning process, which was carried out using the NS + NanoSpinner Plus Electrospinning Equipment (Inovenso Ltd., Istanbul, Turkey). A total of 100 mg of AZC and 100 mg of PVP were dissolved in 10 mL of ethanol. The solution flow rate was 2 mL/h, the high voltage was set at 27 kV, and the distance between the syringe and the aluminum foil-covered rotating collector was established at 12 cm, and a needle of the size 20 G was used. The studies were conducted with no more than 40% humidity and at 25 °C.

#### 2.2.1. Scanning Electron Microscopy (SEM)

SEM images were performed using a scanning electron microscope (Quanta 250 FEG, FEI, Waltham, MA, USA) to assess the morphology of prepared nanoparticles. Before analysis, the nanofibers were sputter-coated with gold palladium.

#### 2.2.2. X-Ray Powder Diffraction (XRPD)

Using X-ray diffraction equipment (Panalytical Empyrean, Almelo, The Netherlands) and a copper anode (CuKα—1.54 Å), the resulting nanoparticles were assessed in a crystallographic assay. The Bragg–Brentano reflection mode configuration was used for the measurements, and the parameters were set at 40 mA and 45 kV. With a step size of 0.05° and a measurement time of 45 s each step, the measurement range was established as being between 3° and 60°.

#### 2.2.3. Solubility Studies

Solubility studies of AZC were carried out in water. The saturated solutions of the drug were prepared by adding an excess of the drug to the liquid vehicle (about 200 mg per 1 mL) kept on the orbital shaker for 48 h at 25 °C. The solutions were diluted, and their concentrations were analyzed using the HPLC-DAD method. An LC system with Chromeleon software version 7.0 (Dionex Thermoline Fisher Scientific) was used as HPLC measurement equipment. A 250 mm × 4 mm LiChrospher RP-18 column with a 5 μm particle size was used for the separations (Merck, Germany). A diode array detector operating at a maximum wavelength (*λ*_max_) of 210 nm was used to make the detection. The mobile phase consisted of buffer (4.6 g of monobasic potassium phosphate anhydrous dissolved in 900 mL of water, adjusted with 1 N sodium hydroxide to a pH of 7.5, and diluted with water to 1 L) and acetonitrile in a ratio 35:65% *v*/*v*. The column temperature was kept at 40 °C, and the mobile phase flow rate was 2.0 mL/min.

### 2.3. Preparation of AZC-Based Soft Hydrogels

Based on the Design of Experiment (DoE) data and a 3^2^ full factorial design (Statistica 13.3 software, TIBCO Software Inc., Palo Alto, CA, USA), the hydrogels’ composition (amount of chitosan and degree of chitosan deacetylation) was chosen and is shown in Table 1.

The soft hydrogels were made by weighing water in a beaker (20 mL), adding the AZC-based nanoparticles (corresponding to 1% AZC content, 200 mg, in the hydrogel mass) and appropriate CS mass (200 mg for 1%, 400 mg for 2% and 600 mg for 3%), according to the Table 1, and stirring for five minutes at 500 rpm. After that, 1% acetic acid (200 μL) was applied to dissolve the chitosan. Before testing, the soft hydrogel was agitated for twenty-four hours. After preparation, the hydrogels were stored at room temperature.

The *n* value, release of AZC, antioxidant activity (as measured by the DPPH method), anti-inflammatory activity (as indicated by the degree of hyaluronidase enzyme inhibition), and bioadhesive properties were the parameters used to evaluate the effectiveness of soft hydrogel production.

### 2.4. Characteristics of Soft Hydrogels

#### 2.4.1. Fourier Transform Infrared Spectroscopy with Attenuated Total Reflectance (ATR-FTIR)

ATR-FTIR spectra were recorded using an IRTracer-100 spectrophotometer (Shimadzu, Kyoto, Japan) in absorbance mode over a 400–4000 cm⁻^1^ spectral range. The instrument was set to a resolution of 4 cm⁻^1^ with 400 scans and utilized Happ–Genzel apodization. Data processing was carried out using LabSolutions IR software (version 1.86 SP2, Shimadzu, Kyoto, Japan).

#### 2.4.2. Rheology

Basic rheological characterization was carried out using a Physica MCR 301 rotational rheometer (Anton Paar, Graz, Austria) equipped with a sandblasted plate-plate measuring system (PP25/S). Flow curves were obtained in the shear rate range $\dot{\gamma }$ = 0.01–100 s^−1^ at a temperature of 25 °C. Viscoelastic properties were determined using oscillatory measurements: amplitude sweep tests (γ = 0.01–1000%, ω = 5 rad/s) and angular frequency sweep tests (ω = 0.01–100 rad/s, γ = 1%) at 37 °C.

#### 2.4.3. Diffusion Tests

Vertical Franz diffusion cells (PermeGear, Inc., Hellertown, PA, USA) were used to conduct in vitro release tests on the soft hydrogels. Each cell held 5 mL of acceptor solution (artificial saliva solution: potassium chloride (1.20 g), sodium chloride (0.85 g), di-potassium hydrogen orthophosphate (0.35 g), magnesium chloride (0.05 g), calcium chloride (0.20 g), xylitol (20.0 g) and water up to 1 L; pH was adjusted to 6.8 by 1 M HCl). Regenerated cellulose membranes (Nalo Cellulose^®^, Kalle GmbH, Wiesbaden, Germany) with pore diameters of about 25 Å were attached to the cells. Prior to the experiment, the membranes were submerged in the acceptor fluid for 24 h at 37.0 ± 0.5 °C. The donor compartment was filled with 1.0 mL of gel samples distributed equally across the artificial membrane’s surface. The cells used have an effective diffusion area of 0.64 cm^2^. During the test, the temperature of the receptor fluid was maintained at 37.0 ± 0.5 °C, and it was agitated at 400 rpm. At the proper intervals, samples (2.0 mL) were removed from the acceptor compartment and promptly replaced with an equivalent volume of brand-new acceptor fluid. The HPLC-DAD method described above was used to ascertain the AZC concentrations in the gathered samples.

#### 2.4.4. Biological Activity

*Antioxidant Activity*

The antioxidant activity was determined by using an assay with 2,2-Diphenyl-1-picrylhydrazyl (DPPH). The procedure has been described previously [21]. In brief, 25 μL of hydrogel and 175 μL of DPPH solution (0.2 mmol/l DPPH) were combined and incubated for 30 min in the dark at room temperature. Absorbance was measured at 517 nm in comparison to the blank sample (25 μL of the water and 175 μL of methanol) (Multiskan GO 1510, Thermo Fisher Scientific, Vantaa, Finland).

*Anti-Hyaluronidase activity*

The procedure of hyaluronidase inhibition was determined by a turbidimetric method described previously [21]. Briefly, 25.0 µL of incubation buffer, 25.0 µL of hyaluronidase enzyme solution (30 U/mL), 10.0 µL of the tested hydrogels, and 15.0 µL of acetate buffer were mixed in the well. After 15 min of incubation (37 °C) with shaking, 25.0 µL of hyaluronic acid (HA) solution was added and incubated for 45 min with shaking (37 °C). After this time, 200.0 µL of CTAB solution in 2% sodium hydroxide was added. After 10 min of incubation without shaking (at room temperature), the absorbance (λ = 600 nm) was measured using a plate reader (Multiskan GO 1510, Thermo Fisher Scientific, Vantaa, Finland).

#### 2.4.5. Microbiological Activity

The microdilution method was used to determine the minimal inhibitory concentrations (MIC). The study utilized the *Streptococcus mutans* ATCC 25175 strain and clinical strains of methicillin-sensitive *Staphylococcus aureus* and vancomycin-resistant *Enterococcus faecium* (VRE). Cultures were grown in tryptic soy broth (TSB, Graso Biotech, Starogard Gdański, Poland) on 96-well plates (Nest Scientific Biotechnology, Jiangsu, China), with a final volume of 100 µL per well, achieving a final inoculum concentration of 10⁶ CFU/mL in each well. The bacterial suspension was prepared following McFarland standards. Serial dilutions of each hydrogel were prepared, beginning at a concentration of 20 mg/mL. Further methodological details are available in our previous publication [22]. The plates were incubated at 36 °C for 24 h. After incubation, MIC values were determined visually or, additionally, by a color reaction following the addition of 10 µL of a 1% aqueous solution of 2,3,5-triphenyl tetrazolium chloride (TTC) (Sigma Aldrich, Poznań, Poland).

#### 2.4.6. Mucoadhesive Properties

A viscometric method was used to predict bioadhesive properties [23]. The bioadhesive binding strength between mucin and hydrogels was measured using a viscometric technique, and the viscosity of the prepared hydrogels was measured using an AMETEK Brookfield DV2T viscometer (Hadamar-Steinbach, Germany).

The viscosity coefficient of a hydrophilic dispersion, including mucin and bioadhesive polymers HPMC and Carbopol^®^, was computed using the equation below:

η_t_ = η_m_ + η_p_ + η_b_

where η_t_ is the viscosity coefficient of the system, and η_m_ and η_p_ are the individual viscosity coefficients of mucin and bioadhesive polymer, respectively, and η_b_ is the viscosity of the component due to bioadhesion.

### 2.5. Statistical Analysis

The statistical analysis was carried out using Statistica 13.3. The ANOVA test and Tukey’s post hoc range test for multiple comparisons were used to examine the variances between the mean values and Duncan’s post hoc test. At *p* < 0.05. differences across groups were deemed significant. Principal component analysis (PCA) and PQStat Software Version 1.8.4.142 (2022) were used to analyze correlations.

## 3. Results and Discussion

### 3.1. Preparation of AZC-Based Nanoparticles

According to the biopharmaceutics classification system (BCS), WHO classified azithromycin as a BCS class II (poor solubility) or IV (poor solubility and permeability) compound [3]. Low water solubility limits the formulation possibilities of AZC; therefore, a process for obtaining nanoparticles in a form with potentially improved solubility properties was developed.

**Figure 1:**
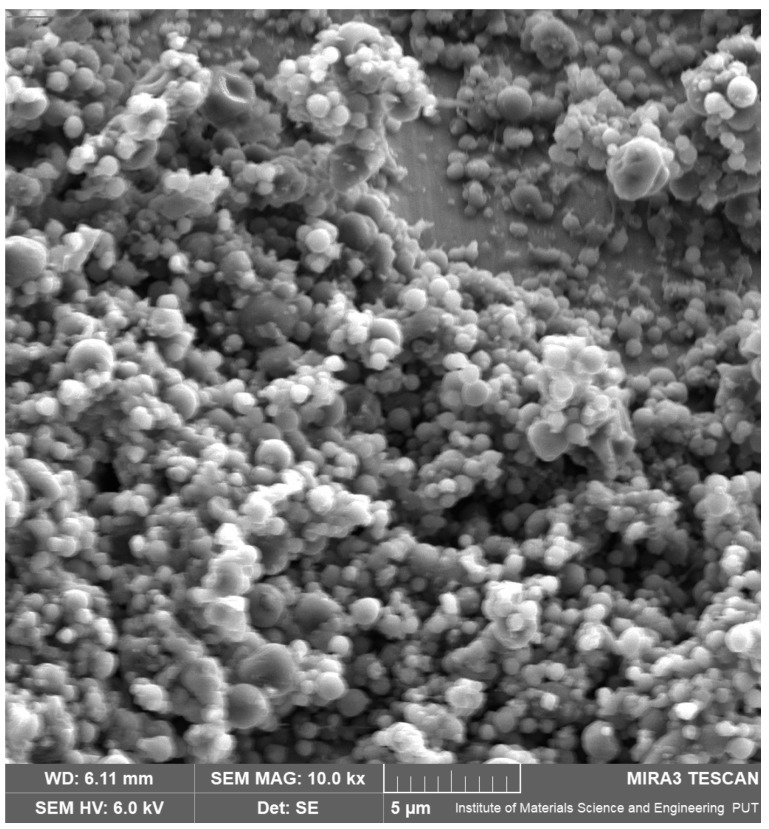
SEM image of AZC-based nanoparticles.

The AZC-based nanoparticles were successfully synthesized using an electrospinning process. The surface morphology of the nanoparticles was examined by SEM (Figure 1), which revealed that all particles were discrete entities, slightly spherical with a smooth surface and a diameter below 1 μm.

**Figure 2:**
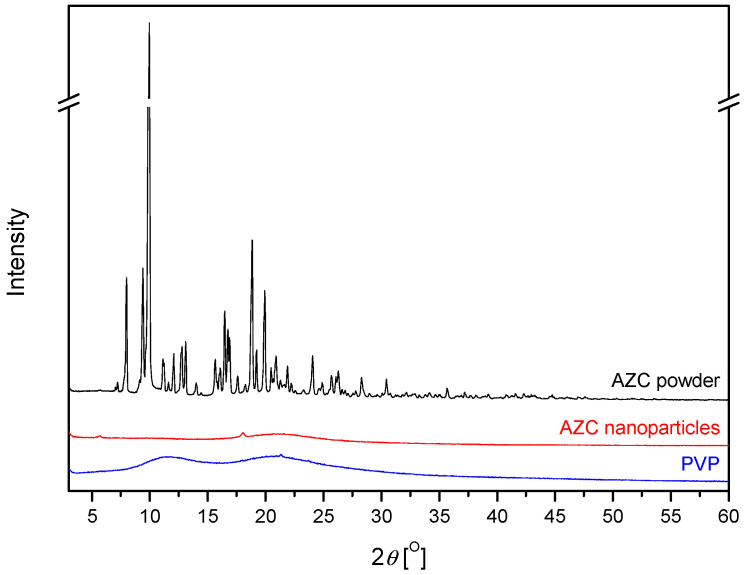
XRPD Diffractogram of AZC powder and nanoparticles and PVP.

The characteristic X-ray diffraction pattern of AZC showed that the main peak occurred in 2*θ* about 10° and other important peaks appeared at 2*θ* about 9.5°, 12°, 15.5°, 16.5°, 17.5°, and 18.8° [24], which confirmed the crystalline nature of the AZC powder (Figure 2). The PVP diffraction pattern shows a large broadening of the diffraction peaks at low intensity, which indicates the amorphous structure of the substance. In contrast, the XRPD of AZC-based nanoparticles did not show any distinctive peaks for native AZC, which revealed that the drug was in an amorphous state.

Finally, the solubility of AZC was assessed. While for AZC powder, the solubility was 2.40 ± 0.05 mg/mL [25], and for nanoparticles, it was 105.3 ± 0.06 mg/mL (n = 3), achieving more than 40-fold improvement in solubility. This is a higher solubility than previous attempts by obtaining urea-based solid dispersions [26] in single and binary micellar mediums [27] or by preparing an amorphous form by quenching cooling of the melt [28].

### 3.2. Preparation of AZC-Based Soft Hydrogels

Because of the high improvement in AZC solubility, starting the formulation work on hydrogels was possible. These nanoparticles were subsequently incorporated into hydrogels prepared with varying degrees of chitosan deacetylation (DDA) and concentration based on a factorial experimental design (Table 1). Thus, all 9 hydrogels were successfully synthesized.

### 3.3. Characteristics of Soft Hydrogels

**Figure 3:**
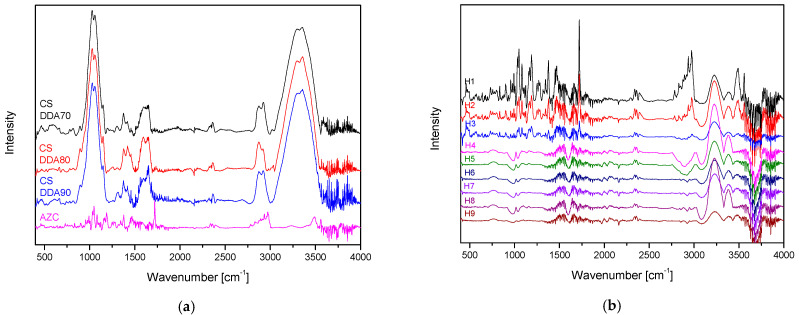
FTIR-ATR spectra of 3 types of CS and AZC (**a**), and hydrogels H1–H9 (**b**).

To verify the successful grafting of the AZC-nanoparticles onto CS, the FTIR-ATR spectra of neat CSs, the AZC (Figure 3a), and hydrogels H1–H9 were recorded (Figure 3b). It is easy to identify the characteristic absorption bands in the CS spectrum: strong absorption bands at 1150 cm^−1^ and 1059 cm^−1^ due to the asymmetric stretching of the C-O-C bond and the C-O stretching vibration of secondary alcohols, respectively; a broad band around 3300 cm^−1^ attributed to the overlapped peaks of the O-H and N-H stretching vibrations; and the amide I (C=O stretching) and amide II (N-H bending) modes at 1638 cm^−1^ and 1578 cm^−1^ respectively [29]. In the case of the AZC spectrum, the following major bands were identified: bands related to the axial stretching and bending of C-H of the methyl groups, at 2750–3020 cm^−1^ and 1377 cm^−1^; the sharp and intense band located at 1719 cm^−1^, assigned to the axial stretching of the C=O group present in the lactone; bands observed in the range of 1134–1221 cm^−1^, appeared due to the absorption associated to the axial stretching of C-O [30].

These characteristic peaks were also found in the hydrogel spectra. A low-intensity peak at 2750–3020 cm^−1^, which becomes less intense with increasing CS concentration and increasing DDA. The above indicates that the hydrogels were successfully synthesized.

Mechanical characterization of the soft hydrogels and assessment of the rheological properties were carried out based on flow curves and basic oscillation tests. Table 2 presents the parameters determined by the power-law model. On their basis, the shear-thinning nature of the tested systems was clearly confirmed; in all cases, the characteristic flow index *n* was lower than 1 (statistically significantly reduced by CS concentration; Figure S2, Supplementary Materials). At constant DDA, the flow index decreased with increasing polymer concentration, resulting from the greater share of long polymer chains that were unwinding and ordering along the shear field. At the same time, a lesser effect of DDA on the change of the *n* coefficient was noted, and it is closely related to the polymer concentration. The consistency coefficient *k*, as expected, increases significantly with increasing biopolymer concentration; again, the highest effect of DDA is observed for the highest polymer concentration (Figure S3, Supplementary Materials). From the point of view of a potential injection application, the best system would be the H6 medium, which has the lowest flow index *n*. However, to confirm these assumptions and due to the relatively high value of the consistency coefficient *k*, an in-depth analysis should be performed using instrumental injectability tests [31] and capillary rheometry techniques.

**Figure 4:**
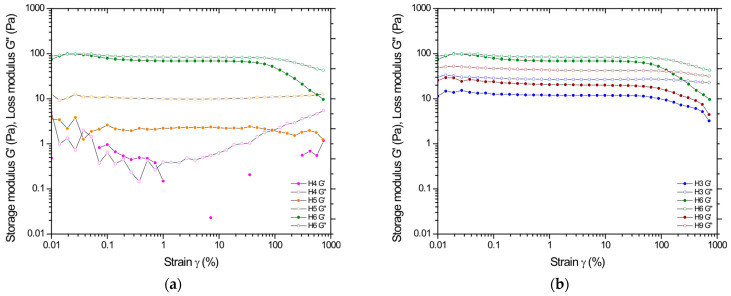
Amplitude at constant DDA = 80% and variable CS concentration (samples H4–H6) (**a**), and then for formulation based on 3% CS and variable DDA (samples H3, H6, H9) (**b**).

To determine the viscoelastic properties and assess the mechanical state of the polymer matrix, frequency sweep tests, so-called mechanical spectra, were carried out, preceded by determining the linear viscoelastic region in amplitude sweep tests at 37 °C. Figure 4 shows the dependence of the dynamic moduli on the applied amplitude (results for all formulations in Figure S1 in Supplementary Materials). In all cases, a predominance of viscous features over elastic ones was observed, represented by a higher value of the loss modulus G″ than the storage modulus G′. Again, the strong influence of polymer concentration on the range in which a linear response is observed under the applied deformation is evident here. Irrespective of the degree of deacetylation, at the lowest polymer concentration (H1, H4, H7), the storage modulus G′ reaches unmeasurable values (at the sensitivity limit of the instrument); therefore, these samples were not subjected to frequency sweep tests. Based on the changes in moduli values outside the LVE range, it can be concluded that the studied systems exhibit the strain-thinning LAOS behavior typical for most polymer solutions, resulting from the chain orientation or order of microstructures along the flow direction [32].

**Figure 5:**
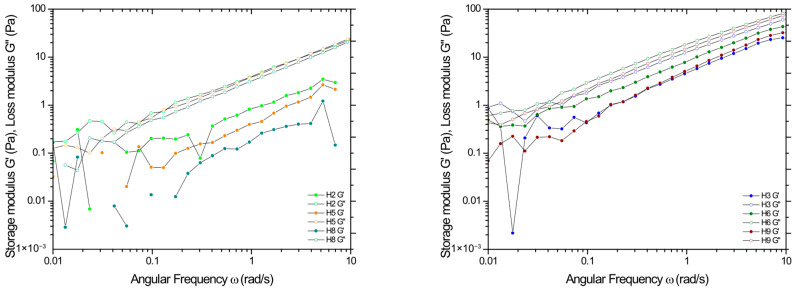
Angular frequency sweep studies of hydrogels based on 2% CS and 3% CS.

Figure 5 shows the mechanical spectra of the 2% and 3% formulations. In all cases, viscous properties again dominate over elastic properties (G′ > G″) over the entire range of angular frequencies used. Additionally, the power-law nature of the dependence of both modules (*n*~0.75) on frequency indicates that the studied systems are in the region of molecular flow [33]. The highest modulus values are achieved by formulations H6, H9, and H3, respectively, which correlates with the results obtained from amplitude sweep tests and flow curves. It would seem, therefore, that from a purely mechanical point of view, these systems should be referred to as soft hydrogels [34] or even structural liquids [35] and are undoubtedly polymeric sols [36] characterized by relatively long relaxation times and, thus, limited flow ability. For this reason, these systems are characterized by sufficient mechanical properties for the intended applications where the formulation is not required to transfer stresses but only to trap the drug substance, which is possible due to relatively high viscous responses.

**Figure 6:**
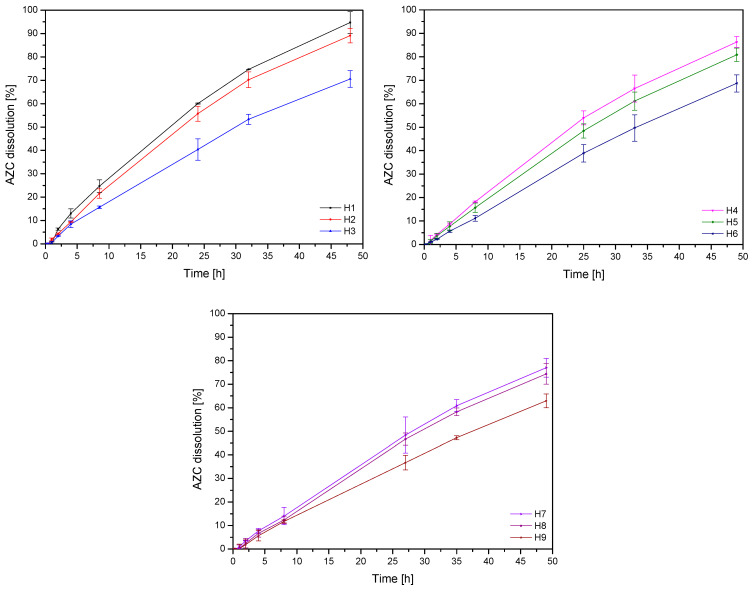
Dissolution profiles of AZC from hydrogels H1–H9 (n = 3).

In vitro release studies were performed to evaluate the dissolution behavior of azithromycin from hydrogels H1-H9. In vitro dissolution studies demonstrated that the release of AZC was significantly influenced by the hydrogel composition, with dissolution percentages ranging from approximately 62.9% (H9) to 94.7% (H1) after 48 h (Figure 6, Table 3). The CS content and CS DDA had a statistically significant effect on the amount of released substance; with increasing concentrations of CS and CS DDA, the release rate decreased (Figure S4, Supplementary Materials). Higher CS concentrations result in a denser hydrogel network, which restricts the movement of drug molecules and slows their diffusion through the matrix into the surrounding medium. This densification is further enhanced by the increased crosslinking and network formation of chitosan [37]. Also, this is confirmed by the increase in the consistency coefficient from rheological tests; higher viscosity means more difficult diffusion due to the increase in medium resistance. The interactions, such as hydrogen bonding and ionic interactions, are intensified in the presence of higher chitosan content, creating a tighter and more compact gel structure that acts as a barrier to drug release [38].

Additionally, hydrogels with higher CS concentrations exhibit prolonged swelling or erosion dynamics. Critical to the drug release mechanism, these processes are slowed in denser hydrogels, resulting in a sustained release profile [39]. Conversely, a higher DDA indicates a more significant proportion of free amino groups (-NH_2_) on the CS polymer, resulting in enhanced protonation of these groups in acidic environments [40]. This increase in positive charge leads to stronger ionic interactions between chitosan and the negatively charged AZC molecules, effectively binding the drug more tightly to the hydrogel matrix and reducing its release rate. Additionally, the increased DDA enhances the ability of CS to form a denser and more cohesive hydrogel network. The higher concentration of amino groups promotes stronger hydrogen bonding and electrostatic interactions within the polymer chains, leading to a more compact and less porous structure. This denser network restricts the diffusion of azithromycin molecules, thereby slowing their release from the hydrogel [41]. These combined effects—stronger drug-polymer interactions, thicker and less permeable network, and slower matrix dynamics—result in controlled and sustained release of AZC, highlighting the ability to control drug release kinetics through precise adjustments in hydrogel formulation.

Mathematical modeling of the drug release profiles confirmed controlled-release behavior across all hydrogels. Models such as zero-order and the Korsmeyer-Peppas best fit most formulations (Table 4). The good fit of the Korsmeyer–Peppas model likely indicates a combination of diffusion-governed AZC release through the hydrogel matrix and physical changes in the hydrogel, such as swelling or erosion. In this case, the combination of the two models indicates a constant release rate over time, suggesting a controlled and sustained drug release mechanism, which is particularly desirable for maintaining constant therapeutic levels of the drug over an extended period without the need for frequent administration.

Biological activity tests confirmed the hydrogels’ effectiveness (Table 5). Both antioxidant and anti-inflammatory properties improved with higher CS content and DDA CS (Figures S5 and S6, Supplementary Materials).

The antioxidant activity of chitosan is primarily attributed to its free amino groups (-NH_2_), which act as proton donors to neutralize free radicals like DPPH. When the concentration of CS increases, the number of amino groups in the hydrogel rises, providing more active sites for radical scavenging. Similarly, an increased DDA corresponds to a higher proportion of deacetylated units in the polymer, meaning more free amino groups are available [42]. The improved density and accessibility of these functional groups enhance the polymer’s ability to interact with and neutralize free radicals, leading to greater antioxidant activity. Furthermore, high-DDA chitosan’s enhanced hydrogen-bonding capability and chelation properties may stabilize reactive oxygen species, further contributing to its antioxidant effects. Similar conclusions were reached in an earlier study evaluating CS-based local delivery systems [11].

CS’s ability to inhibit hyaluronidase, an enzyme involved in the breakdown of hyaluronic acid during inflammatory responses, is also linked to its amino groups. These groups can interact with the enzyme or substrate, disrupting its activity [43]. As the chitosan concentration increases, the abundance of amino groups available for such interactions increases, enhancing the enzyme-inhibiting capacity. Additionally, with a higher DDA, the polymer contains more free amino groups, intensifying the interaction with hyaluronidase and resulting in more potent inhibition of its activity [11]. Moreover, the increased mucoadhesion and density of high-concentration and high-DDA chitosan hydrogels may prolong the retention time at the site of inflammation, allowing sustained activity against enzymes like hyaluronidase. This structural characteristic ensures that the bioactive components of chitosan remain in contact with the enzyme for a longer duration, amplifying the anti-inflammatory effects.

The synergy between the increased concentration and higher DDA of chitosan results in a dual enhancement of antioxidant and anti-inflammatory activities. Both properties are underpinned by the abundance and reactivity of the free amino groups, which serve as the primary functional units for neutralizing radicals and inhibiting enzymes. These improvements make high-concentration, high-DDA chitosan formulations particularly effective for applications requiring oxidative stress mitigation and anti-inflammatory effects, such as in wound healing and oral care therapies.

**Figure 7:**
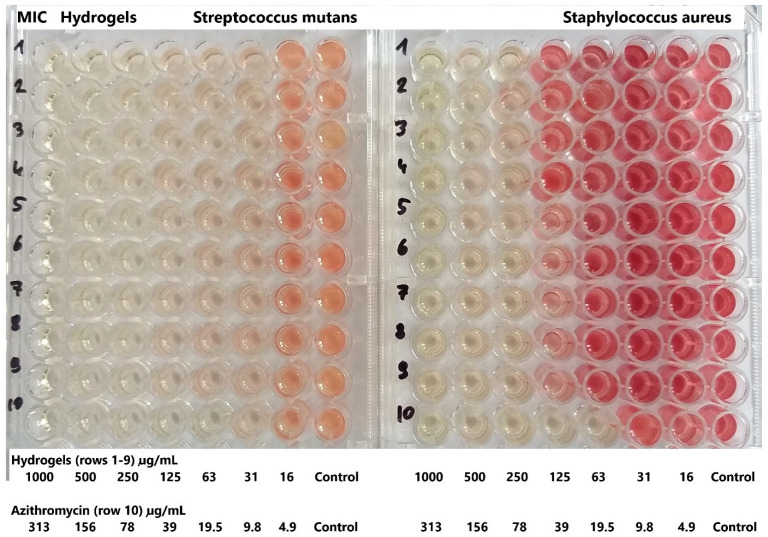
An example image of a 96-well plate from the minimal inhibitory concentrations (MIC) study. Wells stained with TTC as pink, red, or burgundy indicate bacterial growth. The numbers represent: 1—hydrogel H1, 2—H2, 3—H3, 4—H4, 5—H5, 6—H6, 7—H7, 8—H8, 9—H9, 10—AZC (n = 3).

The studies demonstrated very good activity of all hydrogels against *Streptococcus mutans*, with a MIC level of 31 µg/mL, and good activity against *Staphylococcus aureus*, with MIC levels of 125–250 µg/mL (Figure 7). *Streptococcus mutans* is a well-known cariogenic bacterium, meaning it is directly involved in developing dental caries (cavities). *Staphylococcus aureus*, although more commonly associated with skin and soft tissue infections, can occur in the oral cavity and has been associated with various oro-pharyngeal infections, including localized oral abscesses. The MIC for AZC was 9.8–19.5 µg/mL. At the same time, the hydrogels showed no activity against vancomycin-resistant *Enterococcus faecium* (VRE). An example photo of the plate is shown in Figure 7. So, the hydrogels tested in these studies show promising antimicrobial activity against key oral pathogens that are major contributors to tooth decay and other oral infections. This highlights the potential of such hydrogels to improve oral hygiene practices and prevent infections, ultimately contributing to better oral health outcomes.

The mucoadhesive properties of the hydrogels were also a focus, as these are critical for effective dental applications. The hydrogels are intended to be applied from a tube and applied directly to the gum/mucosa of the oral cavity; in this case, the form must demonstrate mucoadhesive properties and stick to the mucosa because it is exposed to oral factors, such as saliva flow. Hydrogels with higher chitosan content and DDA demonstrated enhanced bioadhesion (Table 6; Figure S7, Supplementary Materials). The mucoadhesive nature of CS increases with its concentration due to the abundance of amino groups available for interactions with mucin or water molecules [44]. This enhanced mucoadhesion contributes to a stronger water-holding capacity of the hydrogel, creating a more hydrated yet less permeable environment, further impeding the diffusion of AZC. This makes them suitable for prolonged retention at the application site, enhancing therapeutic efficacy.

**Figure 8:**
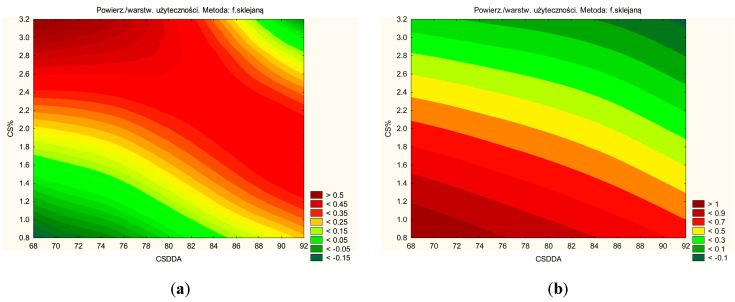
Prediction for effect with a positive sign like dissolution, antioxidant activity, a component of bioadhesion (**a**) and negative sign like *n* value and anti-inflammatory activity (**b**).

The DoE model (Figure 8) was successfully predicted through experimental work and statistical analysis. The optimal soft hydrogel composition was determined to be 3% CS with 80% DDA.

**Figure 9:**
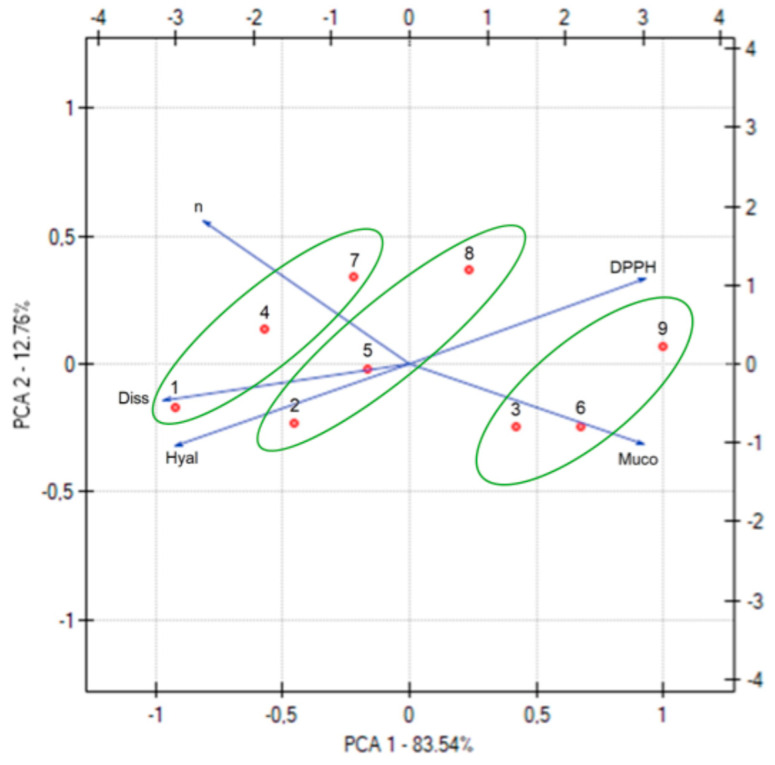
PCA analysis of soft hydrogels properties. The numbers represent: 1—hydrogel H1, 2—H2, 3—H3, 4—H4, 5—H5, 6—H6, 7—H7, 8—H8, 9—H9).

Principal Component Analysis (PCA) was performed to indicate correlations between soft hydrogels’ properties (Figure 9; Table S1, Supplementary Materials). PCA identifies patterns and relationships among multiple interdependent variables while reducing data complexity. Unlike traditional correlation analysis, which examines pairwise relationships, PCA provides a holistic view by transforming correlated variables into independent principal components [45]. This approach helps reveal underlying trends, such as trade-offs between drug release, mucoadhesion, and biological activity, making it particularly useful for optimizing multifactorial formulations like hydrogels. PCA revealed a strong positive correlation between AZC release and anti-inflammatory activity and a negative correlation with antioxidant activity. In all cases, these properties are primarily attributed to the free amino groups, as previously described. Additionally, a strong correlation was observed between AZC release and mucoadhesive properties. Specifically, as the mucoadhesion strength increases, AZC release from the hydrogels decreases.

The soft hydrogels can also be categorized based on their CS content (green lines in Figure 8). Hydrogels with a low CS content, such as H1, H4, and H7, are characterized by a higher percentage of substance release and higher *n* value (hydrogels with a lower CS content will be easier to squeeze out of the tube). The higher percentage of released substance is due to lower viscosity and medium resistance. In contrast, hydrogels with a high CS content, including H3, H6, and H9, exhibit superior mucoadhesive properties.

Overall, the hydrogels demonstrated promising characteristics for dental drug delivery, effectively combining sustained drug release, bioadhesion, and biological activity. Notably, their strong mucoadhesive properties allow them to remain at the application site for an extended period, representing a significant improvement over conventional oral rinses available on the market, which are quickly cleared from the oral cavity.

However, further optimization to scale up production and clinical evaluation are recommended to confirm their potential for practical application. Exploring the hydrogels’ effectiveness in in vivo models will provide crucial insights into their performance under physiological conditions, including biocompatibility, safety, and efficacy. These studies could also identify potential long-term effects, such as tissue response or degradation kinetics. With continued innovation, these hydrogels could become a cornerstone in advanced dental therapeutics, improving patient outcomes and oral health care strategies.

## 4. Conclusions

This study successfully developed AZC-based soft hydrogels with promising potential for dental drug delivery. AZC nanoparticles, synthesized via electrospinning, showed a 40-fold solubility improvement and were incorporated into soft hydrogels with varying chitosan (CS) concentrations and degrees of deacetylation (DDA). The optimal composition was determined to be 3% CS with 80% DDA, as predicted by the DoE model. These soft hydrogels demonstrated controlled, sustained drug release, influenced by CS content and DDA, with denser networks slowing drug diffusion.

These findings introduce a novel strategy for dental drug delivery by integrating AZC nanoparticles with chitosan-based soft hydrogels, offering a unique combination of sustained release, enhanced mucoadhesion, and multifunctional bioactivity. Unlike conventional dental formulations, this system achieves a 40-fold increase in AZC solubility through electrospinning and optimizes drug diffusion by tailoring chitosan content and deacetylation degree. This innovative approach ensures prolonged therapeutic action and enhances antimicrobial, anti-inflammatory, and antioxidant properties, making it a breakthrough in localized oral treatments.

With further optimization and clinical validation, these hydrogels can redefine dental therapeutics by improving drug stability, efficacy, and patient compliance. They could be developed into advanced oral gels, patches, or injectable formulations for treating periodontitis, dental caries, and post-surgical healing, offering a versatile and effective alternative to conventional dental treatments.

## Figures and Tables

**Table 1:** Factorial soft hydrogel preparation experiment plan.

No.	CS DDA	CS %
H1	70	1
H2	70	2
H3	70	3
H4	80	1
H5	80	2
H6	80	3
H7	90	1
H8	90	2
H9	90	3

**Table 2:** Power-law model parameters of formulations H1-H9 at 25 °C.

No.	n [-]	k [Pa^.^s^n^]	R^2^
H1	0.97	0.37	0.9990
H2	0.85	4.42	0.9933
H3	0.72	21.43	0.9873
H4	0.99	0.29	0.9992
H5	0.86	7.04	0.9905
H6	0.68	37.93	0.9854
H7	0.98	0.39	0.9993
H8	0.89	5.43	0.9939
H9	0.76	23.97	0.9867

**Table 3:** AZC dissolution at 48 h (n = 3).

No.	AZC Dissolution at 48 h [%]
H1	94.72 ± 4.72 ^a^
H2	89.11 ± 3.10 ^a,b^
H3	70.57 ± 3.56 ^e,f^
H4	86.33 ± 2.32 ^b,c^
H5	80.88 ± 3.87 ^c,d^
H6	68.67 ± 3.67 ^f,g^
H7	76.99 ± 3.98 ^d,e^
H8	74.37 ± 4.37 ^d,e,f^
H9	62.94 ± 2.94 ^g^

Mean values within a column with the same letter are not significantly different at *p* = 0.05 using Duncan’s test.

**Table 4:** Parameters of mathematical models fitted to the AZC release profiles from hydrogels H1-H9.

Hydrogel	Mathematical Model							
No.	Zero-Order Kinetic		First-Order Kinetic		Higuchi Kinetic		Korsmeyer-Peppas Kinetic	
	K	R^2^	K	R^2^	K	R^2^	R^2^	n
H1	**2.10**	**0.98**	0.10	0.67	9.22	0.77	**0.81**	**0.57**
H2	**1.98**	**0.98**	0.09	0.76	8.58	0.75	**0.93**	**0.54**
H3	**1.54**	**0.99**	0.09	0.74	6.60	0.75	**0.84**	**0.52**
H4	**1.91**	**0.99**	0.09	0.77	8.21	0.74	**0.92**	**0.53**
H5	**1.78**	**0.99**	0.09	0.79	7.56	0.73	**0.91**	**0.52**
H6	**1.49**	**0.99**	0.09	0.83	6.23	0.71	**0.88**	**0.49**
H7	**1.73**	**0.99**	0.09	0.79	7.35	0.73	**0.89**	**0.52**
H8	**1.68**	**0.99**	0.10	0.75	7.06	0.72	**0.78**	**0.52**
H9	**1.39**	**0.99**	0.10	0.72	5.85	0.72	**0.71**	**0.51**

The best fit to the model is bolded.

**Table 5:** Antioxidant and anti-hyaluronidase activity of hydrogels H1–H9 (n = 9).

No.	Antioxidant Activity [%]	Anti-Inflammatory Activity IC_50_ [mg Hydrogel/mL]
H1	10.96 ± 1.25 ^f^	44.71 ± 3.61 ^a^
H2	12.35 ± 1.52 ^e,f^	30.91 ± 2.80 ^b^
H3	17.99 ± 2.04 ^b,c,d^	20.39 ± 1.92 ^c^
H4	13.69 ± 0.80 ^e^	30.34 ± 3.80 ^b^
H5	16.37 ± 1.79 ^d^	27.49 ± 0.97 ^b^
H6	18.87 ± 1.14 ^b,c^	11.67 ± 2.20 ^d^
H7	16.98 ± 0.64 ^c,d^	22.27 ± 2.82 ^c^
H8	20.39 ± 0.50 ^b^	9.32 ± 0.54 ^d^
H9	24.65 ± 1.54 ^a^	7.49 ± 0.67 ^d^

Mean values within a column with the same letter are not significantly different at *p* = 0.05 using Duncan’s test.

**Table 6:** Component of bioadhesion of hydrogels N1-N9 (n = 3).

No.	Component of Bioadhesion [cP]
H1	125.00 ± 7.07 ^e^
H2	1100.00 ± 28.28 ^d^
H3	3900.00 ± 254.56 ^c^
H4	170.00 ± 14.14 ^e^
H5	1250.00 ± 42.43 ^d^
H6	4840.00 ± 311.13 ^b^
H7	330.00 ± 14.14 ^e^
H8	1300.00 ± 28.28 ^d^
H9	6260.00 ± 28.28 ^a^

Mean values within a column with the same letter are not significantly different at *p* = 0.05 using Duncan’s test.

## Data Availability

Data are contained within the presented article or Supplementary Material.

## Supplementary Materials

The following supporting information can be downloaded at https://www.mdpi.com/article/10.3390/pharmaceutics17030304/s1, Figure S1. Amplitude (**a**) and angular frequency sweep studies (**b**) of the tested formulations. Figure S2. Statistical analysis for n values: Pareto plot of standardized effects for n values for hydrogels H1-H9. Figure S3. Statistical analysis for k values: Pareto plot of standardized effects for k values for hydrogels H1-H9. Figure S4. Statistical analysis for dissolution behavior: Pareto plot of standardized effects for azithromycin dissolution at 48 h for hydrogels H1-H9. Figure S5. Statistical analysis for antioxidant activity: Pareto plot of standardized effects for DPPH radical scavenging percentage by hydrogels H1–H9. Figure S6. Statistical analysis for anti-inflammatory activity: Pareto plot of standardized effects for inhibition of hyaluronidase enzyme activity by hydrogels H1-H9. Figure S7. Statistical analysis for a component of mucoadhesion: Pareto plot of standardized effects for mucoadhesive properties of hydrogels H1-H9. Table S1. Correlation matrix for hydrogels’ properties.
