# Supplementary material for: Azithromycin-Loaded Nanoparticles Incorporated in Chitosan-Based Soft Hydrogels: A Novel Approach for Dental Drug Delivery
Source: Pharmaceutics. 2025 Feb 26;17(3):304. doi: 10.3390/pharmaceutics17030304 (PMC11945840; doi:10.3390/pharmaceutics17030304)
Supplement: Supplementary file 1 [file pharmaceutics-17-00304-s001.zip › pharmaceutics-3481956-supplementary.pdf]

Azithromycin-Loaded Nanoparticles Incorporated in Chitosan-Based Soft Hydrogels: A Novel Approach for Dental Drug Delivery

Supplementary material

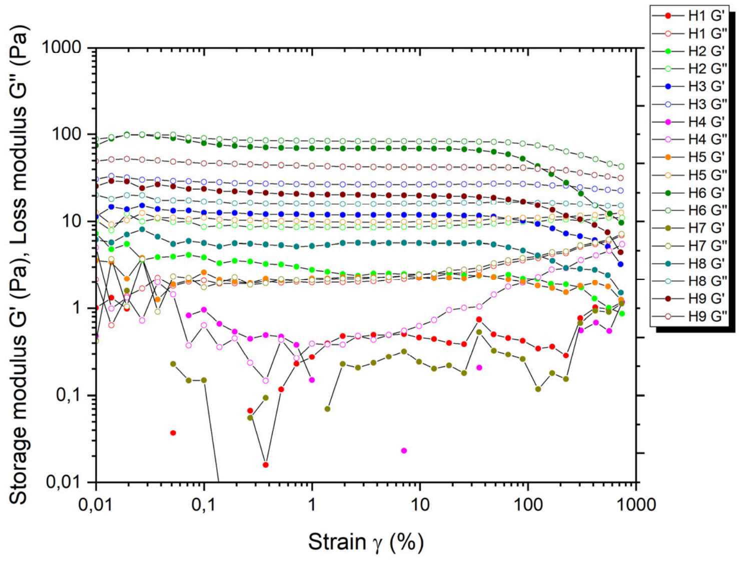

(a)

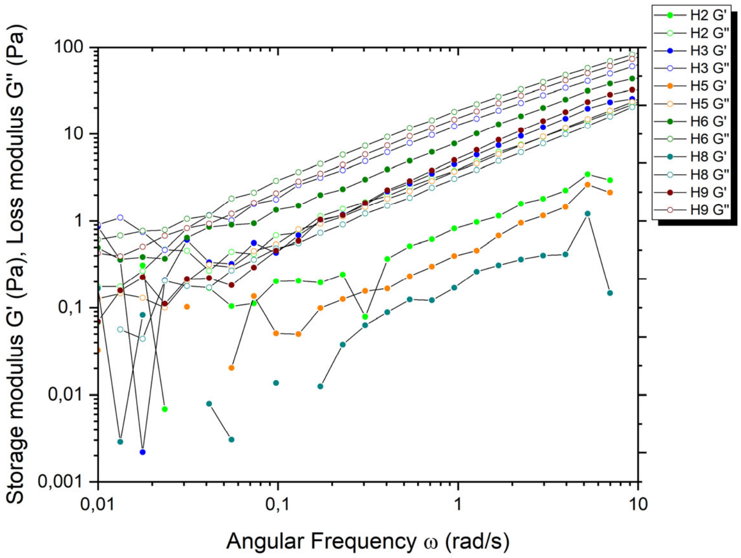

(b)

Figure S1. Amplitude (a) and angular frequency sweep studies (b) of the tested formulations.

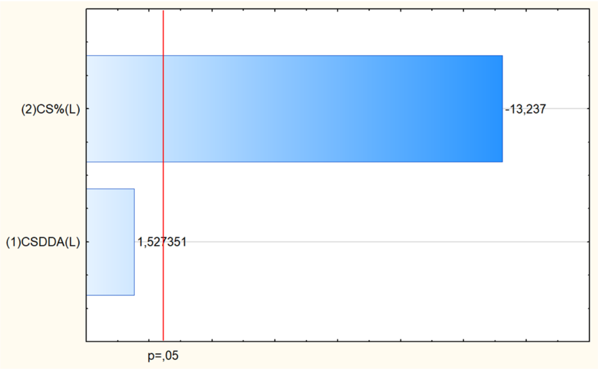

Figure S2. Statistical analysis for *n* values: Pareto plot of standardized effects for *n* values for hydrogels H1-H9.

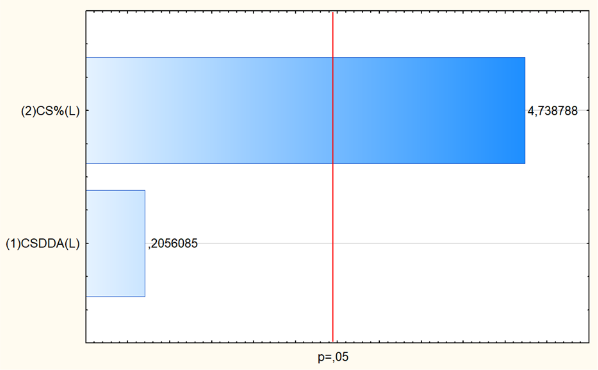

Figure S3. Statistical analysis for *k* values: Pareto plot of standardized effects for *k* values for hydrogels H1-H9.

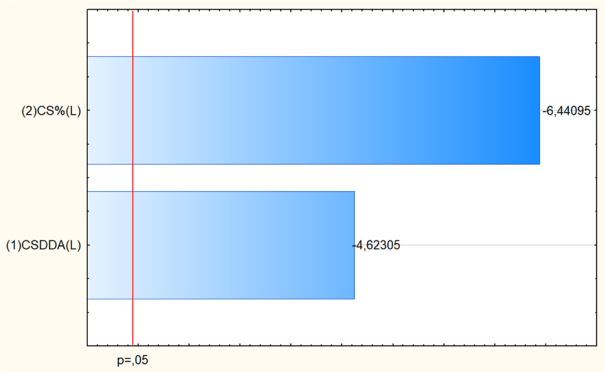

Figure S4. Statistical analysis for dissolution behavior: Pareto plot of standardized effects for azithromycin dissolution at 48h for hydrogels H1-H9.

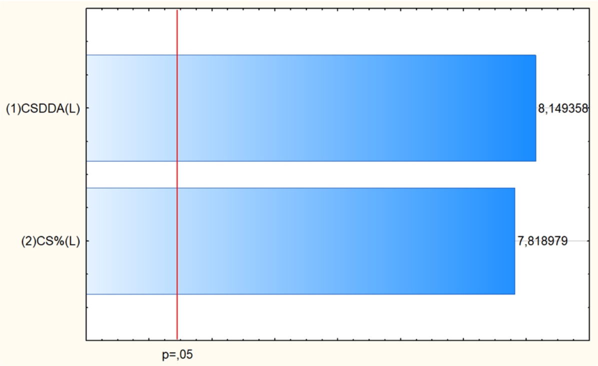

Figure S5. Statistical analysis for antioxidant activity: Pareto plot of standardized effects for DPPH radical scavenging percentage by hydrogels H1-H9.

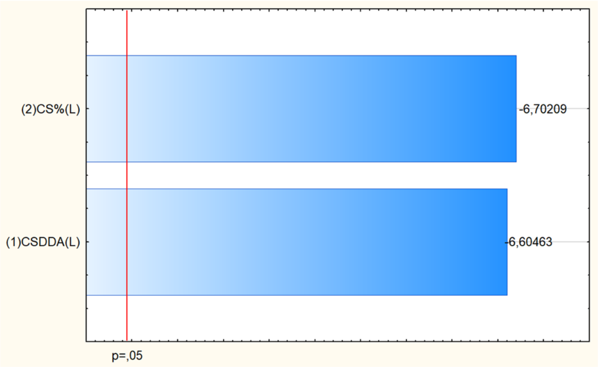

Figure S6. Statistical analysis for anti-inflammatory activity: Pareto plot of standardized effects for inhibition of hyaluronidase enzyme activity by hydrogels H1-H9.

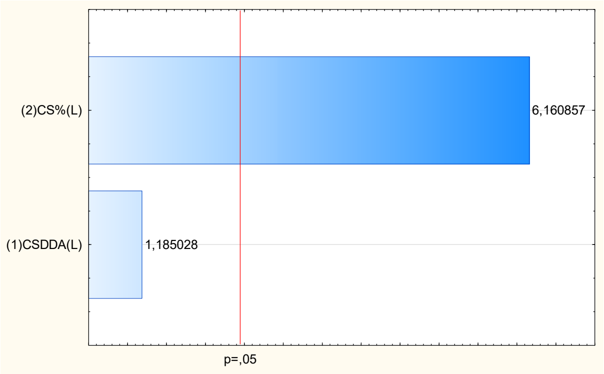

Figure S7. Statistical analysis for component of mucoadhesion: Pareto plot of standardized effects for mucoadhesive properties of hydrogels H1-H9.

Table S1. Correlation matrix for hydrogels' properties

Variable	n value	AZC dissolution	DPPH	Hyal	Component of mucoadhesion
n value	1	0.7120	-0.5608	0.6017	-0.8867

AZC dissolution	0.7120	1	-0.9480	0.9297	-0.8470
DPPH	-0.5608	-0.9480	1	-0.9356	0.7743
Hyal	0.6017	0.9297	-0.9356	1	-0.7138
Component of mucoadhesion	-0.8867	-0.8470	0.7743	-0.7138	1
